# Sociocultural determinants of nomadic women’s utilization of assisted childbirth in Gossi, Mali: a qualitative study

**DOI:** 10.1186/s12884-018-2027-3

**Published:** 2018-10-03

**Authors:** M.A. Ag Ahmed, L. Hamelin-Brabant, M.P. Gagnon

**Affiliations:** 1 Université Laval, 1050 Avenue de la Médecine, room 3696, Québec, G1V 0A6 Canada; 2 Faculty of Nursing Sciences, Université Laval, 1050 Avenue de la Médecine, room 3447, Québec, G1V 0A6 Canada; 3 Faculty of Nursing Sciences, Université Laval, 1050 Avenue de la Médecine, room 1426, Québec, G1V 0A6 Canada

## Abstract

**Background:**

In sub-Saharan Africa (SSA), nomads account for 30 to 60 million people. Their mobility, due to a constant search for pastures and water points, makes health services less accessible to them. Few nomadic women use assisted delivery, which increases the risk of maternal mortality. The reasons behind this limited use have been poorly documented. The objective of this study was to understand the sociocultural determinants of assisted childbirth by nomadic women.

**Methods:**

We conducted a qualitative research in the health area of Gossi (Mali), mainly populated by nomads. Data were collected through a literature review, 26 semi-structured interviews, a non-participant observation, and a logbook. Nomadic women who gave birth in the past three months were included in the study, whether they used assisted delivery or not. A thematic content analysis was performed with QDA Miner software.

**Results:**

The study identified a complex combination of determinants resulting in the use or non-use of assisted childbirth by the nomads of Gossi. Several participants recognized the value of assisted delivery but gave birth at home. They identified sociocultural determinants related to their representations and bodily experiences; the risks and emotions (fear, stress, anxiety) associated with pregnancy; the onset of labor and delivery; and their weak autonomy in terms of movement, decision-making, and economic agency. Nomadic women are not free in their movements, and in order to seek care, they require the permission and support of a man (husband, brother, or father). Furthermore, the participants are housewives, and men control family resources and make decisions regarding all financial matters. Assisted delivery is often only considered when there are complications.

**Conclusion:**

This research has made it possible to understand the sociocultural determinants of the use of assisted childbirth among nomadic women, which should be taken into account when organizing health services for these populations.

## Background

Despite significant progress in reducing its ravages, maternal mortality remains a major concern for public health [1, 2]. Worldwide, the maternal mortality ratio (MMR) fell from 385 to 216 deaths per 100,000 live births between 1990 and 2015, a drop of 44% [3]. However, almost all of these maternal deaths (99%) occur in developing countries (DCs) [4]. Unlike other regions of the world, Sub-Saharan Africa (SSA), with about 62% of global maternal deaths [5], did not experience significant improvement [6]. Moreover, SSA still has the highest MMR in the world, with 546 deaths per 100,000 live births [7], and the World Health Organization (WHO) estimates the number of women living with untreated obstetric fistula to be over 2 million in Asia and SSA [6]. These high maternal mortality rates have severe health and social consequences for the survival of children and for the financial health of the family.

Despite the efforts made, the situation in Mali is not different from that of other SSAs. According to the latest Demographic Health Survey (DHS V), conducted in 2012, the MMR is 368 maternal deaths per 100,000 live births, while the WHO estimated it at 587 per 100,000 live births in 2015 [7]. These figures also mask significant disparities between income levels and between rural and urban populations [8, 9]. In particular, the risk for nomadic women of dying from maternal causes is very high [10].

There are multiple causes of this high maternal mortality [11]. In DCs, obstetric complications are the main cause of maternal death [12]. In fact, they will occur in about 40% of all pregnant women [13] and apparently cause about 80% of their deaths [14]. More than half of these deaths are related to direct causes, such as hemorrhage (27%), infections (10%), hypertension during pregnancy (14%), and abortions (7%) [15]. Next, indirect obstetric causes represent 27% of maternal deaths. It turns out that these obstetric complications cannot be predicted [16]. All women are therefore at risk of developing a life-threatening obstetric complication during pregnancy.

This high maternal mortality is not acceptable because most maternal deaths are due to preventable causes or modifiable behaviors [17, 18]. There is scientific evidence in particular regarding the potential for skilled care at birth as a means of preventing maternal deaths [14]. According to some estimates, it can reduce maternal mortality from 16 to 33% [19], which makes it the most effective intervention to save women’s lives [20].

Furthermore, if it is agreed that assisted childbirth is a relevant strategy for avoiding maternal deaths [21, 22], it is also clear that women do not have the same opportunities to use it because of their sociocultural and economic differences or differences in the places where they live. Indeed, several factors can delay the use of maternal healthcare or make its use impossible [17, 18]. For this reason, despite scientific evidence attesting to the effectiveness of assisted childbirth and the efforts made by several countries to make quality health services available, women continue to give birth at home. In fact, coverage for assisted childbirth is 66% worldwide, but is only 49% for SSA and South Asia [23], compared to 97–99% in high-income countries [24].

For nomadic pastoralists in SSA, the use of healthcare services is very limited when compared to the general population [25]. This is due to several constraints, on the one hand, stemming from their environment and their way of life and, on the other, from important social and spatial disparities. Although underestimated in demographic statistics [26], these nomadic pastoralists are numerous, comprising an estimated 20 to 30 million people in the Sahel [25]. They are defined by Wiese [27] as anyone living in a tent, taking care of livestock, and practicing transhumance. In Mali, nomadic pastoralists are mainly Tuareg, Moor, and Fulani peoples. They live more frequently in low-rainfall desert areas where they migrate throughout the year along well-defined and almost identical routes depending on their rights of access and use, as well as the availability of resources for themselves and their animals [25].

In Mali, the use of assisted childbirth was already limited among nomadic pastoralists, but further deteriorated with the conflicts in the north and center of the country where they live. In fact, in these regions, about 7% of health facilities in Timbuktu, 4% in Gao, and 69% in Kidal were still closed in 2016 due to the security situation [28]. Assisted childbirth proportions in Mali, while reaching 58.4% for the whole country, decrease to 2.6% in Timbuktu, 2.4% in Gao, and 3.3% in Kidal [29]. However, even before the outbreak of war in northern Mali in 2009, these proportions were 35% for Timbuktu, 29% for Gao, and 29% for Kidal, compared to 66% at the national level. In SSA, these trends are confirmed for nomads. In Tanzania, assisted childbirth rates among nomadic women of Dagota were lower than those of the sedentary Iraqw tribe [30]. Similarly, in Ethiopia, of 478 Afar nomadic mothers interviewed, 83.3% gave birth to their last child at home without skilled care [31].

The reasons why these nomadic women do not use assisted childbirth seem difficult to identify as they vary from one context to another. Several authors in SSA refer to determining factors, which could be geographical, economic, cultural, technical, social, or political [10, 27, 32–34], whereas only a few studies explore them for Mali in particular. However, some studies, whose results will be discussed later, have been carried out in Mali [35], Chad [36], Ethiopia [31, 37–40], Kenya [41], and Sudan [42]. These studies have identified some sociocultural, geographic, and financial determinants that could limit the use of maternal healthcare by nomadic women.

The objective of this study is therefore to contribute to this very limited knowledge in order to improve the use of assisted childbirth by nomads in Mali. More specifically, it aims to describe and reach an insight into the sociocultural determinants of the use of assisted childbirth by nomadic women in the commune of Gossi.

This study was guided by the conceptual model of determinants of delivery service use proposed by Gabrysch and Campbell [43], who were themselves inspired by that of Thaddeus and Maine [44], which seems to be the most operational and best-suited model to understand the determinants of the use of assisted childbirth. Furthermore, it is adapted to the context of DCs [45]. It has the advantage of taking into account not only the risks of complications, but also the normal situations of using maternal care (preventive for instance). For these authors, the factors involved in the decision to use preventive maternal care significantly differ from those determining research in emergency obstetric care in the case of complications. In the second case, the severity of the complication may prevail over cost or distance considerations. In the event of life-threatening danger, even those who consider that normal delivery does not justify expenses and travel to a health center may attempt to overcome these barriers, despite high costs [43]. However, for normal delivery or preventive care, the time might be longer and the decision-making mechanism different in such cases.

In addition, this framework seems to distinguish intention to use care from behavior, i.e. actual use of care. For Gabrysch and Campbell [43], four groups of factors influence intention and behavior with respect to seeking care in a health institution. They may be divided into two groups: sociocultural characteristics and perceived benefits/needs, which influence the intention to use care; and economic accessibility (affordability) and physical accessibility, which influence behavior. However, all these factors interact with one another. In this study, we are more specifically interested in the sociocultural characteristics pertaining to the use of assisted childbirth. However, these sociocultural determinants are not the only ones that influence the use of assisted delivery by nomads. They constitute the first part of the results of the doctoral thesis of the lead author (MAAA). The second part of our results will be presented in another publication and will focus on the determinants related to nomads’ perceptions of the quality and geographical and financial accessibility of health services.

## Methods and study setting

The commune of Gossi (Timbuktu region) was selected as the setting for this research. It encompasses a total surface area of 15,000 km^2^ for a population of 24,065 inhabitants, of whom 90% are nomads [46]. It is mainly inhabited by Tamasheq (or Tuareg) and Fulani [47]. The choice of this commune was based on its characteristics (nomadic); the presence of a functional health center (Kaigourou); and the feasibility of research (security conditions for accessing nomads).

### Study design, population, and sampling

The research design is qualitative, which is particularly apt for describing, explaining, and analyzing complex phenomena that have been little explored. The approach used is similar to ethnography. However, we did not have sufficient time of immersion and direct observation that requires an ethnographic approach. The study population includes nomadic women living in their camps who have given birth during the last 3 months preceding data collection, in order to minimize memory bias. Tamasheq nomads were chosen due to the fact that they form the largest group in the commune and that we are fluent in the local language, which facilitates interview administration and data analysis. A mixture of purposive and convenience sampling techniques was used. Interviews were conducted with women until saturation [48]. To diversify our sample [49], we included women of all ages and with varying parities, who gave birth in health facilities or elsewhere, and whose camps were located at a limited distance from the closest health center.

In preparation for recruitment, we identified the position and movements of nomadic camps in the commune. Then, during eight field trips to four cardinal points, we reached these camps in their pasture areas for four to seven-day stays. Given our limited financial means and security conditions, all trips were within an 80-km radius of Gossi. Once in a camp, we first contacted the camp leader, introducing ourselves, explaining our goals, and asking if there were women who had given birth in the last 3 months. When a woman was identified, we camped nearby and the recruited assistant contacted the woman to explain our goals and ask her for an interview.

### Data collection

The collection of data for this research project took place in three stages. A one-month preparatory stage made it possible to explain the study to the actors (health services, administrative and political authorities), to identify the pasture areas, and to get an insight into the movements of the nomad camps. Logistic means of collection were also identified. Similarly, a local assistant fluent in the Tamasheq language was recruited and trained to conduct the interviews in order to minimize the researcher’s gender bias. Interview guides were translated into the Tamasheq language and pre-tested. They were then slightly adjusted before being validated by our research directorate. A second two-month phase allowed us to collect data in nomad camps. Then, after the interviews were transcribed, we returned to check their validity with five participants.

*Three collection techniques* were used concomitantly:

*Semi-structured interviews* were conducted individually by a trained research assistant and administered in a quiet place away from family members in order to allow research participants to speak freely and thus prevent possible response biases. After obtaining women’s informed consent, all interviews were recorded and lasted between 42 and 76 min each. An internal validation process was undertaken after each interview by compiling information that was not clear under a list of topics, which were pursued more extensively in the next interviews. This technique was suggested as a means of improving the quality and rigor of the interview guide [50]**.**

The guide consisted of open questions based on the conceptual framework provided by Gabrysch and Campbell [43] as well as our review of the literature and was designed to identify the factors determining use of assisted childbirth by the nomads. The guide was flexible and provided the relevant points to be addressed. The questions of the first part of the interview guide focused on the socio-demographic characteristics of the participants. Then, the other sections discussed the representations and experiences of women during pregnancy and delivery, their use of care, their access to information, their knowledge about health services and the decision-making process for assisted childbirth.

*Non-participant observation* helped us to get an insight into the context while not being directly involved, thus maintaining an external perspective. In order to carry out these observation procedures, we designed an observation grid [51]. The observation sites were nomadic camps. Although the observation sites and procedures were not hidden, we tried to remain as discreet as possible, while taking notes so as not to miss anything. We noted the different stages of the fieldwork (dates, people met, etc.) along with the situations encountered (what the nomads do and the nature of their interactions, etc.), and developed a preliminary interpretation of the phenomena observed as a prelude to data analysis.

We also kept *a logbook* aimed at minimizing our influence on the collection and analysis of data by stimulating our reflexivity and capacity to become aware of our own feelings and biases [52]. The logbook thus helped clarify our positions and ensure greater transparency in the process by recording the way we made decisions throughout the research project.

### Data analysis

Thematic content analysis was used as it is particularly indicated for exploratory and explicative approaches [53]. It describes the material collected and explores its meaning to reflect what the participants said in the most objective and reliable way possible. The analysis started during data translation and transcription. Interviews were first translated in French (language of the thesis) by us, and then in English (for this article) thanks to the services of a professional translator. To ensure that we accurately reported what our participants said, we went back to the field to check the accuracy of the transcripts with five of them.

The verbatim reports were then re-read using the audio recordings in order to validate them and thus ensure the reliability of opinions. This also made it possible to better understand and become familiar with the content of the interviews and separate substantial information from anecdotal elements [54]. This step enabled us to identify the first themes [53] in order to design a draft analysis grid. Next, we began to codify and categorize the data based on inter-coder agreements concerning four interviews, reached with our research supervisor in order to ensure the validity of our analysis. Initially, verbatim were examined line by line and paragraph by paragraph to generate label codes attached to analysis units of variable size. These codes made it possible to decipher the different sociocultural determinants of the use of assisted childbirth by nomads. This coding step is intended to facilitate the manipulation of the data. Once this coding step was completed, the thematization (or categorization) phase started, which consisted of grouping several close codes under a same sub-theme. This process made it possible to group similar answers under the same generic title. Once all the sub-themes were obtained, they were in turn grouped into themes.

We used QDA Miner analysis software, to which the verbatim report was exported in order to facilitate data management. Data were then categorized favoring a type of sequenced approach [53] using both inductively derived and pre-existing themes. First, using an inductive approach, a reading of the verbatim led to the creation of sub-themes and themes. Then, in a second step, they were put into perspective with those predefined through our conceptual framework to build an analysis grid that took into account the important elements reported by participants.

We created a thematic tree representing the hierarchy of themes and sub-themes according to their main or peripheral role to answer our research questions. Their recurrence allowed to have a synthetic representation of the analyzed content in relation to the different cases identified in our study [53]. Thus, thematic groupings (which may be convergent, divergent or complementary) have emerged as kinds of matrices of meaning that coexist with each other. They made it possible to name the different dimensions of the sociocultural determinants. Relationships were then established to find links between these dimensions, and contrasted to form a universe of meaning that allowed us to describe and understand the different sociocultural determinants of the use of assisted childbirth by nomads.

During this process, several steps have been taken to ensure the validity and reliability of our data and analyses. Our interview guide was pre-tested and then adjusted before being validated by the thesis supervisors to ensure that it met our research objectives. The use of the triangulation technique by juxtaposing collection methods and the diversification of sources of information also ensured the validity of our results. Our good knowledge of the field allowed us to understand participants’ language and values ​​in order to minimize interpretative biases that may arise from cultural differences between researchers and participants. Our results have also been compared with those of similar studies on the determinants of health service utilization in SSA. Moreover, we tried to be as transparent as possible throughout the research process. We provided a detailed description of the methods and procedures of our study and continued to improve them as we progressed in our research. Although we are aware that neutrality is impossible in qualitative research, we have undertaken a honest and reflexive approach by reporting in the logbook the different methodological approaches on the ground and recognizing our biases.

## Results

The results are presented in two parts: the sociodemographic characteristics of the participants and the sociocultural determinants of the utilization of assisted childbirth.

### Sociodemographic characteristics of participants

We visited 35 nomadic camps and recruited 26 women. These women were found in 24 camps. The sociodemographic profile of these women includes their place of residence, their age, their occupation as well as that of their husband, and their number of pregnancies. We also noted the use or non-use of prenatal consultation (PNC) and assisted childbirth.

The camps visited are within a radius of 16 to 62 km from the nearest health center (Kaigourou). The study participants are 18 to 40 years old. Their age distribution is as follows: 18 to 25 years (*n* = 11); 26 to 35 years (*n* = 11); and 36 years and older (*n* = 4). Of the 26 participants, 19 (73.1%) claim to have received prenatal care (PNC) at least once at the health center, while only 11 (42.3%) have given birth there during their lifetime. Women in the youngest group report less use of assisted childbirth (7 out of 11 have not had recourse to assisted childbirth).

All the participants are married housewives, and their spouses are breeders. No woman has been to school. The distribution of the number of pregnancies ranges from one to nine pregnancies per woman. Two were primiparous, eight had two or three pregnancies, and 16 had four or more pregnancies. Most of them started to have children at a very young age (11 of the women before the age of 25).

In addition, during 3 months of non-participant observation, we were able to appreciate the social and living environment of the nomads. In the camps we visited, nomads usually live in family (couple and children) under a single tent of leather or cotton of about 20 square meters. The distribution of work within the family seems well codified. The animals are at the center and punctuate nomads’ life. In the daytime, men tend the livestock in the pasture and at night, the whole family gets involved in animal care. When milking is finished and the milk is distributed, families meet to exchange information around a fire. We participated in these exchanges, which, for the occasion, concerned the difficulties of resorting to health services. On the other hand, the life of nomads seems well organized. Their socioeconomic level is function of their social status but also the number of livestock they have. The elderly and the women are highly respected, which gives them several privileges. Also, their way of life allows nomads to be quite autonomous to the extent that they produce most of what they need to live (milk, meat, butter). Other food is often considered as a supplement or luxury that not everyone can afford. In case of illness, self-medication is used first, sometimes with modern medicines brought from the city. However, nomads seem very open to modern health. On the other hand, nomads perceive themselves as quite vulnerable because their whole economy is dependent on unpredictable rainfall. At the same time, they demonstrate resilience capacities under extreme conditions. These observations allowed us to better understand the sociocultural dimensions that directly or indirectly influenced their use of assisted childbirth.

### Body representations and experiences of pregnancy and childbirth

Our findings show that the use of assisted childbirth is influenced by some dominant representations of their body that women develop during pregnancy, at the beginning of labor, and during childbirth.

#### During pregnancy

For many participants, pregnancy is a normal, even unavoidable process. All eyes in the community are turned toward married women waiting for pregnancy: *“Pregnancy is a normal phenomenon for us women. All women are supposed to get pregnant.*” (Aicha, 22).

According to them, the femininity of women and their status as wives are confirmed only through their ability to get pregnant. But beyond that, the fact of not being able to get pregnant could even create problems within the couple: “*Pregnancies are part of our life as a woman even if they make us tired. Married woman are expected to give birth. The opposite would be very frowned upon and could even be problematic.*” (Ami, 38).

Paradoxically, the participants affirm that their pregnancy is often hidden, especially during the first months, because they feel ashamed of having gotten pregnant insofar as this implies the performance of a sexual act, which is a taboo topic of conversation, even with one’s husband:

*Even if I am pregnant, I cannot talk to people; it is a source of shame otherwise. They must understand by themselves ... I do not tell my husband because I do not know what he may think. I do not know if he is aware of what happened to me; we do not talk about it.* (Lalla, 20)

Several participants admit that the neighbors can imagine that a woman is pregnant when she shows some signs, but this will only be confirmed when there is a change in the parturient’s body (visible belly). Typical subterfuge includes keeping the pregnancy secret for several months, which can delay recourse to maternal care.

Once considered pregnant, the woman is viewed as fragile and vulnerable. As such, she receives special attention and measures including the use of healthcare facilities to protect her and enable completion of pregnancy: “*Once she becomes pregnant, a woman is considered a fragile human being. People pay close attention to her so that the pregnancy will succeed. If we feel that she needs something, we must give it to her very quickly; otherwise she risks aborting.*” (Mariama, 33).

However, women recognize the capacity and limits of their bodies to cope with pregnancy and childbirth, a recognition which determines their use of healthcare. Thus, some participants refer to knowledge or experiences related to their bodies, enabling them to deal with any pregnancy without seeking healthcare. They refer to a body that practices, learns, gets used to, and acquires its own abilities to take on pregnancy and childbirth without any assistance: *“With each pregnancy and childbirth, you learn, and your body gets used to dealing with them.*” (Tahouskat, 23).

In addition, other parturients, without questioning their abilities, recognize the limits of this body, especially when obstetric complications appear. In these cases, using healthcare is envisaged or desired by most participants to lessen their suffering: “*You can suffer from illness at such a level that you cannot stand it anymore. One should then consider going to the health center to receive healthcare.”* (Rokiatou, 30).

#### The beginning of labor

The beginning of labor is also an important moment for the participants. It is marked by several representations that tend to delay the use of care. When labor begins, some participants said they would hide the pains to make sure that this was indeed the beginning of the labor and thus avoid conveying false information to neighboring people: *“I had pain for a day and felt that I should give birth, but I did not want to tell anyone about it until I was sure.*” (Fadimatou, 30) Furthermore, to show their bravery, other women hide the beginning of childbirth as long as possible: *“Our tradition is to hide the birth until the last minute so that the woman shows that she is strong.”* (Fatimata, 27).

Once labor begins and the neighbors are informed, a small group of women come to stay with the parturient woman to support her. These women pray at a distance, but the parturient gives birth without any direct assistance: “*I gave birth on my own. The women were there, but they did not help me. They cannot do anything, nor do they care to do so.”* (Safietou, 20) A woman, more often than not the mother of the parturient, will intervene only to cut the umbilical cord: “*I have always given birth at home with the help of my mother. Her assistance involves waiting until I give birth to cut the cord of the child and that’s all.”* (Zeinabou, 32).

#### Childbirth

Childbirth is feared by nomadic women and their neighbors, as this proverb illustrates: *“Before giving birth, every woman has her two feet hung in her grave until delivery.”* (Zeinabou, 32) Indeed, childbirth is unanimously perceived by our participants as a period of danger, and the outcome is uncertain, entailing a high risk of dying: *“The biggest fear for we women is the sinking of the genitals* (genital prolapses)*.... In addition, we can have miscarriages or even die.*” (Tafa, 22) Furthermore, delivery is sometimes feared because of the pain associated with it, although there are different perceptions of this pain among pregnant women. For many participants, giving birth in pain is natural, self-evident, and transient, making health services less relevant: “*Women give birth every week, and deliveries are naturally painful, but it happens anyway and without the presence of health workers.”* (Mamata, 22) Facing the magnitude of these pains, others have recognized their own limits when it comes to bearing pregnancy and thus say they are more favorable to using healthcare services, of which they recognize the effectiveness: *“Delivery is very painful. Even if it is normal, we need help, and health services are very effective.”* (Mariama, 33).

On the other hand, several emotions, combined with their personal experiences, also seem crucial for these women in deciding whether or not to use assisted childbirth. Women talk about their worries and their fears when they refer to the various consequences of a delivery that went wrong and could affect their womanhood and compromise their union. “*I am always afraid because at each delivery, I can end up with a tedafé* (vesico-vaginal fistula in Tamasheq) *or other problems that can make me infertile.*” (Taliat, 22) Also, experiences of anxiety or stress are conveyed when women recount cases of abnormal childbirth. “*Imagine, when childbirth is impeded, you lose hope of living. You tell yourself it’s all over for you.”* (Ami, 38) Facing these emotions, several participants prefer to turn to assisted childbirth.

Home delivery is the most common recourse for pregnant women, although it is not necessarily the first choice of the majority of participants. It is however a deliberate and assumed choice for some parturient women, who for the most part only know this type of childbirth: “*I prefer to give birth at home.*” (Taliat, 22) Their preference for home deliveries will only change if their lives are threatened or when they are no longer able to make a decision. “*Personally, I would go to the health center only when I have no hope left, when people decide for me.*” (Fatimata, 27) Some justify their preferences with a concern for adhering to a social standard or tradition that recommends giving birth at home: “*For home deliveries, we found our parents doing so and we followed in their footsteps.*” (Lalla, 20).

Assisted childbirth is of interest for the majority of participants, although some women gave birth at home:

*I think it would have been better to give birth at the health center according to what I hear. Personally, I always gave birth at home, but that's because I had no other choice. People here are afraid to give birth at the health center; they do not know how it will happen. It is easier for me and my family to give birth at home.* (Safietou, 20)

Women have found several benefits to assisted childbirth, including shorter labor hours and the lessening of pain: *“When you give birth at the health center, with the drugs, the labor time is shortened, and you suffer less.” *(Khadou, 35) Also, parturient women have confessed that health services are also very effective postpartum: *“Since I gave birth at the health center, I’m doing everything to come back because the conditions are better there. You’re cared for and your child too.”* (Mariama, 33) For these reasons, many women no longer consider giving birth outside of health centers: *“Since I discovered delivery at the health center, I try to come back even when I am living far away.” (*Khadou, 35).

### Risks during pregnancy and childbirth

During our interviews, the women insisted on a vast array of simple and complicated risks to which they are exposed during pregnancy and childbirth and which determine their use of healthcare. They represent these risks in different ways according to their own criteria, with a great variability of interpretations. Almost all parturient women acknowledge having experienced various diseases/risks during their pregnancy and childbirth periods: “*As soon as I get pregnant, I get sick. Abdominal pain with constant vomiting. I have to lie down all the time. During pregnancy and childbirth, we face a lot of risks.”* (Lawal, 38) Others also refer to the ultimate risk, which could be death: “*Risks during childbirth are many and various and may lead to death.*” (Tahousket,23) They report that age and a high number of pregnancies (multiparity) add to these risks.

Given this strong perception of risk and the perceived effectiveness of assisted childbirth, the majority of women seek healthcare services: *“I gave birth at home, but more and more women tend to seek care and to give birth at the health center because it seems really helpful for a quick recovery.”* (Fatimata, 27) However, they turn to healthcare services only when they are facing complications.

### The autonomy of nomadic women

Our findings also highlight three dimensions related to the low autonomy of nomadic women, all three constraining their use of assisted childbirth. These are autonomy of movement, of decision-making, and of economic agency.

### Autonomy of movement

Most participants admitted that they did not have freedom of movement and that their movements were governed by rules. In this regard, a woman traveling alone would not be well perceived, and the authorization of the husband or a family member (father or brother) would be required for the use of assisted childbirth. Indeed, the Muslim woman owes obedience and respect to her husband, and as such, challenging his authority is perceived as a religious transgression: “*We are Muslims; the woman cannot challenge instructions from her husband and travel to the center without his authorization as well.*” (Rokiatou, 30) Moreover, for other participants, this is part of the education received from their parents that should be maintained: “*We have always been taught that this is how it is and how our mothers behaved. We must get these authorizations and follow their example.*”(Assietou, 29).

In addition, for most participants, a second requirement for assisted childbirth is that the parturient women be accompanied by a man. The reasons given are primarily pragmatic. The ailing woman needs sturdy arms to cope with the unsuitable mode of transport on damaged roads: “*To go to the health center, you need to find companions first. How can I travel sick in a transport vehicle without being accompanied by a man, a member of my family? It’s not acceptable.*”(Leila, 24) The company of a man during their evacuation was very much appreciated by the participants. However, men are not always available to accompany them, which sometimes makes it impossible to use assisted childbirth: “*I was sick (childbirth), while no man was available to bring me to the health center. They were all busy with the animals.”* (Salka, 30).

Also, once at the health center, the presence of a man would also be required to make decisions regarding expenses and hospitalization, or act as an intermediary between the woman and the health workers: “*I have spoken very little with health workers. They talk to the man (husband) and give him the papers (prescriptions). Then he pays for the drugs.”* (Fatimata, 27) For the participants, all these are elements that limit their mobility as regards recourse to assisted delivery.

### Decision-making autonomy as regards the use of assisted childbirth

Participants explained a complex decision-making process that follows several stages in which various interactions between different people occur. This process highlights the low autonomy of women as regards decision-making.

### A network of actors controlling the decision-making process

For the participants, decision-making is controlled by a network of actors essentially made up of men. For these women, it is primarily the husbands who would be at the forefront when it comes to making decisions: “*I did not see how I could go to the health center while my husband was on a trip.*” (Taliat, 22) Moreover, the parturient woman’s parents, in whose house she gives birth, would preferably be other important actors: “*When the woman gives birth at her parents’ house, they are the ones who decide. This case is the most common one, especially for young people.*” (Fadimata, 40) Sometimes, decision-making is extended to the community, particularly to the notables and especially to the head of the camp, who manages the problem and proposes solutions to the husband. Indeed, at an advanced stage of complications, it is often badly perceived for the husband to decide alone for his wife without referring to the notables: “*It is the notables of the camp who decide ... it is sometimes badly seen for a man to decide alone for his wife.*” (Leila, 24).

This interdependence among husbands, parents, and relatives is recognized by participants as being very valuable in facilitating decision-making.

In addition, some participants negotiated with their spouses to await their delivery in a family near the health center a few weeks before the pregnancy reached full term: “*For deliveries at the health center, if I’m not too stuck by that time, I will live and wait with a family near the center.*”(Khadou, 35) In these cases, the woman asks permission of the husband, who evaluates the suitability of the request, and the decision is made by mutual agreement of the two spouses. This decision is also facilitated by the presence of host families (parents, acquaintances, or friends).

In other cases, this process of negotiation between the spouses did not result in the use of assisted childbirth because the decision was made late, either due to issues of modesty or because the communication between them was incomplete. Despite the insistence of some parturient women, their husbands felt that there were not enough reasons for them to give birth at the health center, which led to their not using the services:

*Once I tried to talk to my husband about going to the village and giving birth at the health center. But he thinks that I am in a good shape and that my deliveries have always gone well at home. So he did not want me to, and I gave birth at home.* (Safietou, 20)

In most cases, the decision of the husband or parents is final, which limits the power of women to use assisted childbirth.

### When there are complications, the decision is made without the parturient woman’s input

For many participants, the decision-making process is different, and for them childbirth entails other steps when there are obstetric complications. In such cases, their decision is determined by the seriousness of the parturient woman’s state of health, which is itself judged on the basis of two criteria. On the one hand, there is the duration of her extended labor: “*The decision is made when the woman spends several days of labor, and the family realizes that she cannot give birth at home.*” (Fadimatou, 26) On the other hand, there is damage relating to the physical health of the parturient woman (fainting, labor stoppage, etc.):

*I had to pass out before my mother would ask my father to take me to the health center. I do not remember anything. My mother later told me how it went. Fortunately, I arrived at the health center in time.* (Khadou, 35)

This decision-making process is subject to a series of negotiations and consultations and requires consensus among family members: “*The decision is made in a concerted manner. There should be mutual agreement before the decision is reached.*” (Fadimata, 40) As a first step, the parturient’s female assistants assess the seriousness and urgency of the situation and provide guidance for the use of the healthcare center. They then inform the men, who make the decision for her evacuation: “*The women around you will recognize the complications. Then they will go to the men to find solutions.*” (Fatimata, 27) For many participants, decision-making would depend heavily on the values ​​and opinions of their husband or parents (father or brothers).

The majority of parturient women mentioned not having participated in this decision-making process, a reflection of their weak autonomy as regards their recourse to assisted childbirth.

### Economic autonomy: Control of financial resources by men

Our interviews show that all the participants are housewives. They do not perform any paid work, which limits their autonomy to seek care. “*We do not perform any paid work to earn money. We take care of the family, children, and old people. It’s already a lot of work. But how can we have money in our bush here? There is no trade or income-generating activity.”* (Lawal, 36) In addition, participants acknowledged that men control family resources and decide on all financial issues. As such, they are responsible for the health of the family and it is up to them, regardless of their wealth, to cope with the expense of caring for their wives: “*When I’m at my husband’s house, he’s the one who decides and gives me the means to go to the health center for care.*” (Khadou, 35) The payment of expenses related to the care of women is therefore an obligation for men. As a result, the costs of care would sometimes discourage them from sending their women to the health center: “*It is the men who decide about everything. Even if a woman has her property, she cannot decide for herself. Their authorization is therefore required to give birth at the health center.*” (Lalla, 20) Women thus acknowledge that they have limited access to family resources and that their control by men would affect their decision-making power to seek care: “*Maybe if women had their own means, they could decide on their own*!” (Lalla, 20).

## Discussion

This study provides important contextual information on a complex combination of determinants, the result of which is the use or non-use of assisted childbirth by Tamasheq nomads in matters of health in ​​the commune of Gossi in Mali. Most participants indicated that they prefer to give birth in health centers, but this is often impossible due to many constraints related to their sociocultural, health, and economic context. In this difficult environment, the use of assisted childbirth is more often an expression of pragmatic choices by nomads and its limitation is related to several determinants.

In fact, the relationship that nomadic women have with their bodies seems to be one of the important elements in explaining their use of assisted childbirth. It appears that this recourse is largely influenced by certain dominant representations that women have of their bodies, risks, pain, and emotions during pregnancy and childbirth. In this regard, our study is in line with several others in SSA that have achieved similar results [55–65]. These studies emphasize, and sometimes differ on, the links between sociocultural determinants and the use of maternal healthcare. However, for the most part, their findings support the contention that these determinants would deter women from using obstetric care [60].

Also, the results of some studies in nomadic areas in SSA mainly match those of our study, namely how pregnancy is detected, the modesty that it entails, and the hiding of the women’s condition which limits their use of maternal healthcare [31, 35–42]. According to these authors, pregnancy gives women a fragile and vulnerable status, more attention, and privileges. On the other hand, the women of Gossi seem to refer less to traditional healers, marabouts, and birth attendants than others in SSA. However, childbirth is also dreaded by all these women, and the rules regarding delivery in particular are similar. Recourse to healthcare is seen as useful only in the case of complications. These sociocultural barriers to the use of maternal healthcare sometimes seem more ingrained than other obstacles, such as those of a geographical or financial nature [36].

Apart from these studies on nomads, several authors in SSA have shown that the representations that women and the communities in which they live make in relation to pregnancy and childbirth largely guide their behavior in the use of healthcare. For most participants, pregnancy and childbirth are seen as “normal phenomena,” giving them the feeling that their bodies are competent to deal with whatever might arise. As such, they do not need to be provided with healthcare [66]. Therefore, this dominant perception of “normal” pregnancy prevents many women from seeking care for routine deliveries [67]. This is why in many contexts, paradoxically, women with access to care give birth at home. In line with our results, some authors conclude that it is not enough to have accessible care for the use of assisted delivery to be effective [67]. They consider that this recourse would instead depend to a greater extent on the relevance to and appropriation of services by the communities in question. On the other hand, if childbirth is often considered as not requiring the use of healthcare, this is not the case when obstetric complications appear. For many nomadic women in our study, only complications would warrant assisted childbirth. On the other hand, the impact of this late recourse on maternal mortality in obstetric emergencies is questionable given the time required to intervene and save women in distress. Some authors even associate it with a high maternal mortality [67].

In addition, the use of assisted childbirth in the Hausa area (Nigeria), for example, is also limited by the fact that unaided childbirth is seen as an act of bravery and pride [56]. Likewise, it is seen in Uganda as an endurance test to which the woman must submit, and maternal death is seen as an act of fate [62]. However, there seems to be an evolution in these beliefs. Presumably, many of our study participants, including those who have never used assisted childbirth, are increasingly recognizing its benefits, particularly to shorten labor hours and postpartum care. Also, the risks and pain associated with childbirth generate a number of emotions that may trigger a preference for assisted childbirth. On the other hand, nomadic women hide their pregnancy, but not for the same reasons mentioned in some studies in SSA. For the nomads, their condition is hidden because acknowledging the pregnancy would suggest the sexual act, which is a taboo topic of conversation. This is not the case, for example, in Cameroon or Zimbabwe, where women apparently hide their pregnancies to protect themselves from “evil spirits,” including witchcraft, to which they believe themselves more vulnerable [61, 66]. Similarly, in Nigeria, it is because of a belief called “Kunya” (meaning modesty) that the primigravida in particular would hide her pregnancy [56].

In several SSA countries, as in the case of nomadic women in our study, childbirth is often perceived as very risky, and this element of risk would justify going to the health center [68]. Indeed, the combination of fear of suffering or of dying during childbirth would largely justify women’s preferences for assisted childbirth. On the other hand, the happiness related to this process seems better expressed in other countries in SSA [69] than among the nomadic women we met.

Moreover, the analysis of participants’ discourse in our study also revealed a low autonomy of nomadic women, which seems to limit their use of assisted childbirth. This is in line with several other studies in SSA that identify three dimensions to judge the level of autonomy of women with respect to using maternal care [70–74].

These authors evaluated the autonomy of women though their level of mobility, participation in decision-making, and finally their access and control of financial resources. Also, they defined autonomy as the ability of women to take control of resources and decisions that affect them and to act independently from the men or the society in which they live [75]. They agree that in SSA, more independent women are more likely to use maternal care [71]. For some authors, women in SSA do not always have freedom of movement [70], as we have observed with the nomads of Gossi. In fact, the consent of their mothers-in-law or their husbands appears to be required before they can seek care [70]. Without the consent of these agents, recourse to medical care is seen as a lack of respect. Furthermore, since the woman has to pay at the health center, she must discuss this matter with her husband or mother-in-law, which is badly perceived locally. On the other hand, when women have greater freedom of movement, they are more likely to use maternal care [70, 74].

Our results are also consistent with other studies having highlighted the low participation of women in decision-making related to assisted childbirth in SSA [70, 73]. As seen in our study, their lack of decision-making power would largely contribute to late recourse or non-use of healthcare [38]. For example, in Ethiopia, when women ultimately decide where to deliver, they are more likely to give birth in a health center than is the case where others make this decision for them [39, 73]. The same is true in Niger, Burkina Faso, or Zambia, where women with some decision-making autonomy are more likely to give birth in a health facility than women who do not have such power [70, 71, 74]. In these countries, women with a greater degree of autonomy are free to make the decision to seek care and are also better equipped to face the imposed constraints.

Another study in Malawi shows that decision-making processes for the use of maternal healthcare are dominated by the men who make the final decision, especially when it comes to obstetric complications [76]. Thus, faced with the opposition of their relatives, some women could not assert themselves and were unable to force their relations to take measures in favor of seeking care [76]. Moreover, the nomadic women in our study have no source of income, and family resources are controlled by men, which seems to limit their use of assisted childbirth. Studies in SSA indicate that maternal care is less important when expenditures are controlled by other household members [72]. In addition, when women have better control over their financial resources, they can then decide how to use them to pay for care.

Our results also match those of Hampshire [36] who found that most of the nomadic women in her study had limited economic power that did not allow them to cope with health costs without the help of others. Their use of healthcare depends on the availability and the good will of men. Furthermore, achieving access to the husband’s resources is different or variable depending on a woman’s marital status (single, geographically single, or married). However, this individualistic and atomistic conception of the origin of autonomy is subject to a certain degree of criticism [77, 78]. According to this conception, individuals are supposed to be sufficiently independent and rational to make their own decisions [77, 79]. It does not recognize people’s social character based on their relationships with others, their interdependence, and the mutual support that defines human lives [80].

According to several authors, this conception thus offers a poor or an incomplete vision of autonomy [80, 81]. Conversely, they put forward a more relational form of autonomy which asserts that individuals are never independent enough to make decisions alone. This alternative conception of autonomy places the individual in a socially integrated network [82]. Its attributes are relationships with the family, the community, and society, and thus mutual responsibility and interdependence are recognized as playing a major role in any decision-making process. Indeed, for any decision-making, including recourse to maternal care, the mutual responsibility and cooperation of women with spouses, parents, and relatives therefore play a determining role, as noted in our study. As a result, women’s social environment and their relationships are recognized as continually contributing to their autonomy [82].

To illustrate the importance of the social network, Gage said in the same vein that in rural Mali, it is not only where you live that is crucial for the use of maternal care, but also who your neighbors are [83]. This suggests that living close to women who have used maternal care is likely to mean more help offered by neighbors. As such, autonomy analyzed from an “individualistic” angle by several authors in SSA has limitations, particularly for the evaluation of the decision-making power of nomadic women encountered in the commune of Gossi regarding the use of assisted childbirth. Indeed, these nomads are organized within their camp and with their neighbors in a supportive community environment. This interdependence within a social network is crucial for their survival in often hostile environments far from urban centers where health services are located. For these nomadic women, the use of assisted childbirth can be seen as a collective process involving multiple actors who interact and share roles according to certain social standards and at different points in the decision-making process. In analyzing the discourse of our participants, we note that the logic of solidarity and mutual aid seems to have determined their use of assisted childbirth given the inaccessibility of health services for them. They described many roles in their social networks that allow them to seek care and, in particular, to exchange information on the services available, as well as to support and help each other and thus to facilitate such use. This information seems to be well appreciated and often considered as a priority for rapid decision-making as regards the use of assisted childbirth. In addition, studies in other contexts have shown the importance of these social networks and their links to the use of healthcare [84–86]. Some authors argue that social networks are an important source of information [86]. For the nomadic women of our study, their social network, including friends or relatives, seems to play an important role in motivating them to seek care. Pregnant women were able to receive emotional support, counseling, or help with housework to enable them to visit health centers.

Our participants consider that recourse to assisted childbirth depends to a greater extent on the suitability of services provided and their appropriation by the communities. On the other hand, if childbirth is often considered as not requiring the use of healthcare, it is not so when obstetric complications ensue. For many participants, it is not enough to have accessible care for the use of assisted childbirth to be effective [67]. Similarly, other authors show that women are more likely to seek care when their networks are broader and more homogenous, and when the level of solidarity is more important [85]. In other words, a network made up of people who are closely related and with homogeneous family ties is stronger and more reliable, as is the case for the participants in our study. Nomadic women seem to appreciate the fact that they do not feel alone in dealing with complex and difficult decisions that require important means, community mobilization, and, above all, knowledge and initiative, to be shown while they are suffering and not always able to cope. They seem to perceive this solidarity and interdependence as a source of strength, an integral part of their identity, and a component of a strategy of support upon which they can rely in a hostile environment. Therefore, we can question the ability of nomadic women with little education to make decisions alone with respect to seeking care in this difficult environment.

### Limitations

This study is one of the first in Mali to address the determinants of the use of assisted childbirth by nomads. It was conducted in a challenging research environment and with limited resources. However, we have sought to mitigate or minimize the impact of these constraints by applying a suitable and rigorous methodology. We decided to provide space for nomadic women, who are not very accessible, to express their views. However, the views of other family members or health professionals could have enriched the data by providing a more complete picture of the different sociocultural determinants. Moreover, it is not possible to generalize or extrapolate the results of this type of qualitative study beyond the commune of Gossi where the data were collected. Nevertheless, what we uncovered was remarkably similar to the findings of other authors in SSA and reflected the difficulties they too noted in nomadic environments.

Another possible limitation is that the assistant who conducted the interviews is from this area, and despite our explanations and instructions, there is a potential bias of social desirability, that is to say that participants have expressed what they perceive as appropriate or socially desired responses. Similarly, a misinterpretation of the French translations of Tamasheq interviews may lead to a distortion of original meanings or a less nuanced understanding of participants’ perceptions as to why they are or are not using assisted childbirth. To mitigate these risks, we returned to the field to check the validity of our data with some participants. The transcripts were also often cross-referenced with our field notes and any discrepancies were discussed and clarified.

## Conclusion

This study helped to identify and understand a set of sociocultural determinants whose interaction influences the use of assisted childbirth by Tamasheq nomadic women in the health area of the commune ​​of Gossi, Mali. It shows that despite a certain evolution, this recourse stems from pragmatic choices and perceived needs, which themselves are determined, on the one hand, by representations and experiences from pregnancy and childbirth, and on the other, by their lack of decision-making autonomy. Moreover, despite the preference of the majority of participants for assisted childbirth, which is recognized as being effective, many of them continue to give birth at home.

Furthermore, interventions aimed at promoting the use of assisted childbirth by nomads should target these different sociocultural determinants. Such interventions should take into account the relationship that nomadic women have with their bodies and their capacity and limits to cope with pregnancy and childbirth. In addition, they would take into account women’s perceptions of the multiple risks recognized as punctuating their experiences of pregnancy and childbirth, as well as the unpredictable complications that strongly motivate demand for healthcare.

Similarly, the study describes the mobilization of a social capital that must be recognized to the extent that it seems a very valuable component in this decision-making process. Therefore, potential interventions should capitalize on the social network of nomadic women to support decision-making where the use of assisted childbirth is being promoted.

## Abbreviations

DCs: Developing countries
DHS: Demographic Health Survey
Km: Kilometer
MMR: Maternal mortality ratio
PNC: Prenatal consultation
SSA: Sub-Saharan Africa
WHO: World Health Organization
